# Supplementary figures and images for: Volatiles Induced from Hypolepis punctata (Dennstaedtiaceae) by Herbivores Attract Sclomina erinacea (Hemiptera: Reduviidae): Clear Evidence of Indirect Defense in Fern
Source: Insects. 2021 Oct 28;12(11):978. doi: 10.3390/insects12110978 (PMC8618567; doi:10.3390/insects12110978)

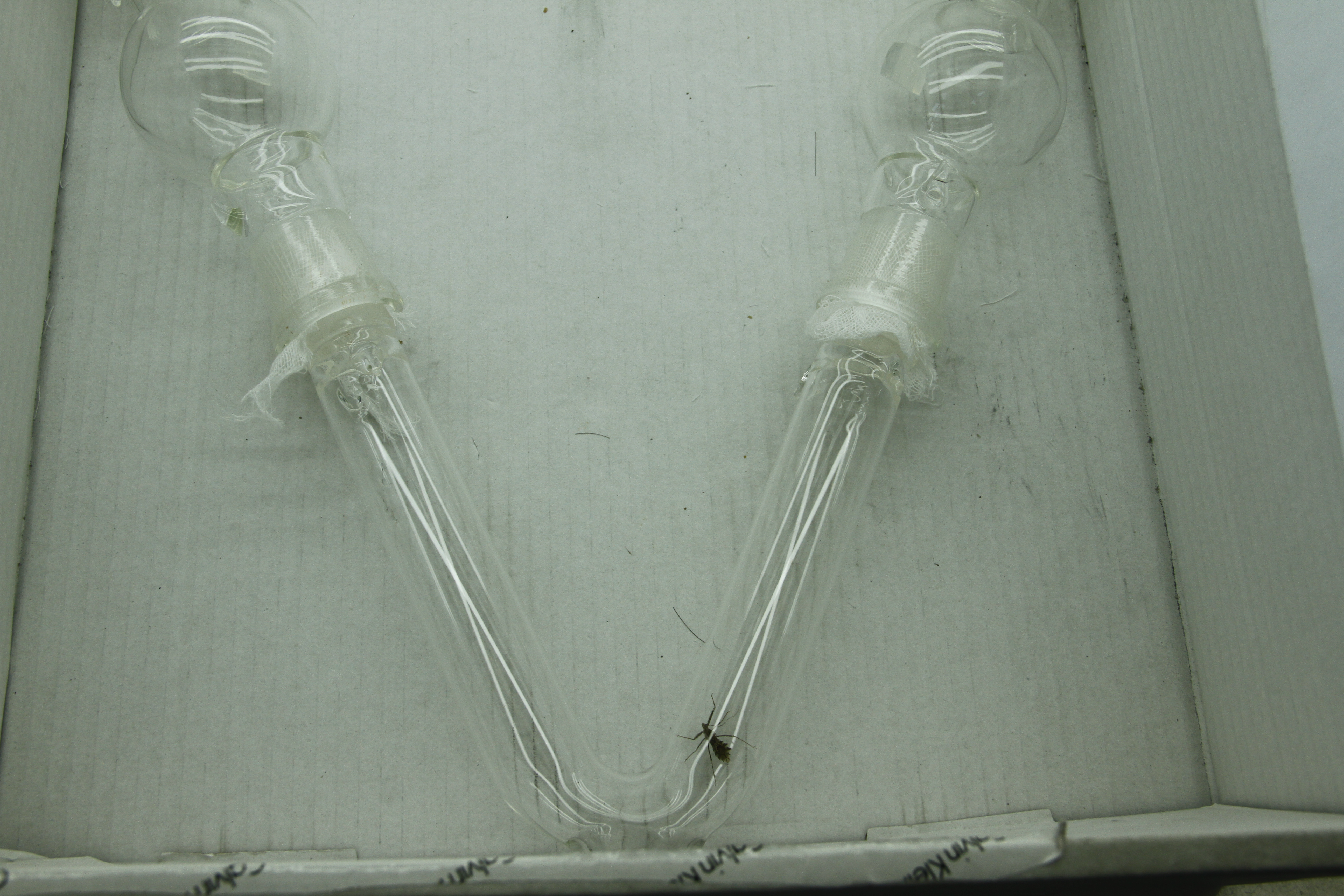

Supplement: Supplementary file 1 [file insects-12-00978-s001.zip › Figure S2.tif]

Sample graph of the Y-olfactometer

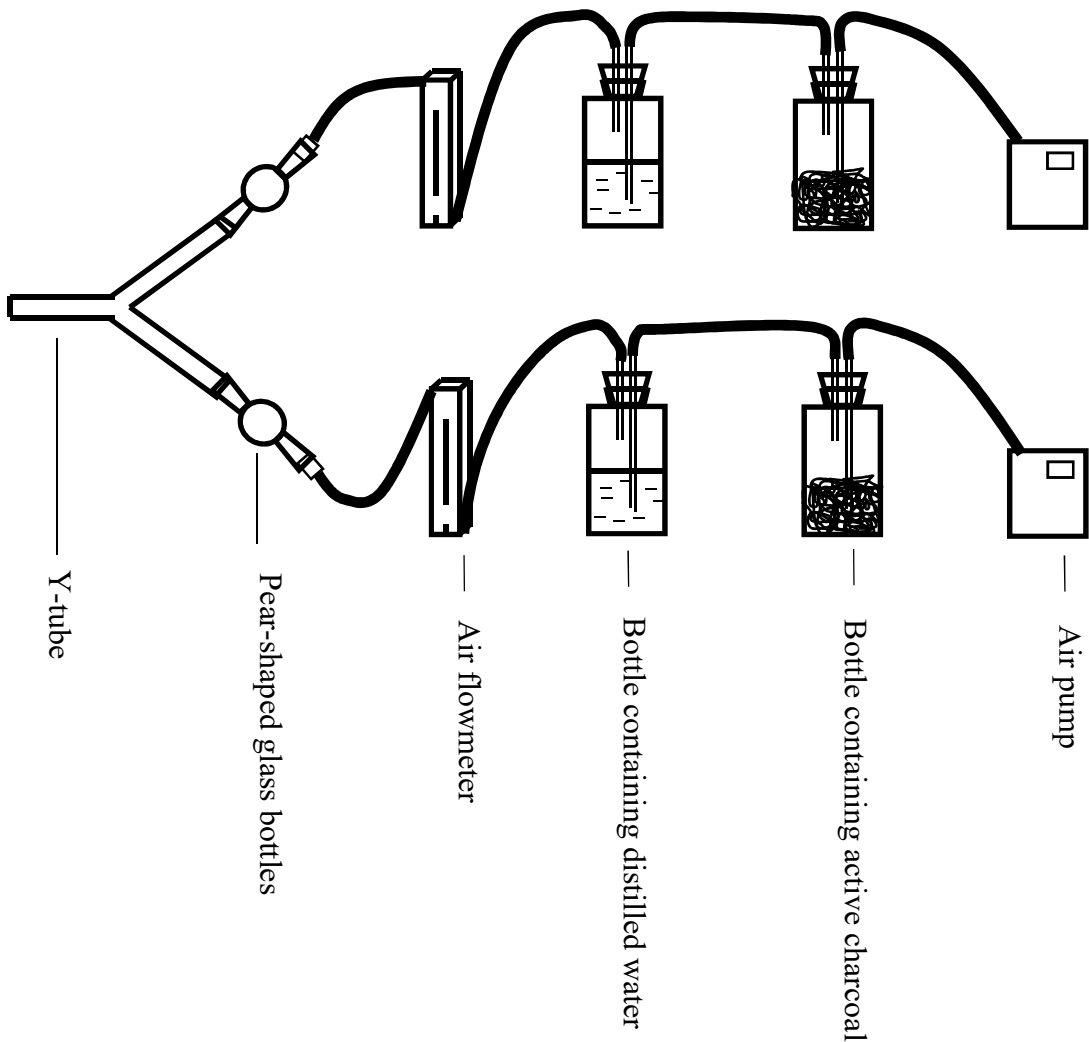

Supplement: Supplementary file 1 [file insects-12-00978-s001.zip › Figure S1.pdf]
